# To Everard Home, Esq.

**Published:** 1806-06

**Authors:** Samuel Young


### To EVERARD HOME, Esq.

Sir,

In Case xxxvii of your "Observations on Cancer," after amputation of the leg had been performed by Mr. Hunter in an instance of "Fungated Sore in the Foot," we are informed, that he was again consulted about three months after, respecting her future management, (at which time the stump had completely healed,) and was then told, that she had a swelling on the upper part of her thigh, near the groin, which she had observed about a week after the amputation, and which had since increased very considerably."

The case then concludes as follows:

"He examined it, and found a swelling of one of the lymphatic glands, about the size of a walnut, prominent under under the skin, and a little painful when pressed. Just above this, and in the groin, there was a smaller one, which appeared likewise to be diseased. These glands, he made not the least doubt, were buboes, in consequence of absorption, from the disease of the foot, contaminated prior to the amputation; and, from taking all circumstances together, he did not advise the extirpation of them. If they had been searched for, and removed, at an earlier period, perhaps at the time of the amputation, or soon after the operation, it is probable that she might have recovered."

Now, Sir, all that is wanted here (and which, as the case at present stands, is impossible to determine) is, simply to ascertain whether the contamination of these glands, rested on your's and Mr. Hunter's opinion; and therefore as such, and from taking all circumstances together, the conclusion is made, that if they had been removed at an earlier period, "she might have recovered?" "Or, whether, on the contrary, this observation arose subsequently to the *opinion of contamination,* in consequence of cancer actually seizing the groin, and from which disease the patient was destroyed?"

As this is a very material point in the history of the case, I am sure, your goodness will not refuse, that information which can alone render it conclusive.

I am, &c.

*March* 1806. SAMUEL YOUNG.

